# A highly efficient multi-core algorithm for clustering extremely large datasets

**DOI:** 10.1186/1471-2105-11-169

**Published:** 2010-04-06

**Authors:** Johann M Kraus, Hans A Kestler

**Affiliations:** 1 Institute of Neural Information Processing, University of Ulm, 89069 Ulm, Germany; 2 Department of Internal Medicine I, University Hospital Ulm, 89081 Ulm, Germany

## Abstract

**Background:**

In recent years, the demand for computational power in computational biology has increased due to rapidly growing data sets from microarray and other high-throughput technologies. This demand is likely to increase. Standard algorithms for analyzing data, such as cluster algorithms, need to be parallelized for fast processing. Unfortunately, most approaches for parallelizing algorithms largely rely on network communication protocols connecting and requiring multiple computers. One answer to this problem is to utilize the intrinsic capabilities in current multi-core hardware to distribute the tasks among the different cores of one computer.

**Results:**

We introduce a multi-core parallelization of the k-means and k-modes cluster algorithms based on the design principles of transactional memory for clustering gene expression microarray type data and categorial SNP data. Our new shared memory parallel algorithms show to be highly efficient. We demonstrate their computational power and show their utility in cluster stability and sensitivity analysis employing repeated runs with slightly changed parameters. Computation speed of our Java based algorithm was increased by a factor of 10 for large data sets while preserving computational accuracy compared to single-core implementations and a recently published network based parallelization.

**Conclusions:**

Most desktop computers and even notebooks provide at least dual-core processors. Our multi-core algorithms show that using modern algorithmic concepts, parallelization makes it possible to perform even such laborious tasks as cluster sensitivity and cluster number estimation on the laboratory computer.

## Background

The advent of high-throughput methods to life sciences has increased the need for computer-intensive applications to analyze large data sets in the laboratory. Currently, the field of bioinformatics is confronted with data sets containing thousands of samples and up to millions of features, e.g. gene expression arrays and genome-wide association studies using single nucleotide polymorphism (SNP) chips. To explore these data sets that are too large for manual analysis, machine learning methods are employed [1]. Among them, cluster algorithms partition objects into different groups that have similar characteristics. These methods have already become a valuable tool to detect associations between combinations of SNP markers and diseases and for the selection of tag SNPs [2,3]. Not only here, the size of the generated data sets has grown up to 1000000 markers per chip. The demand for performing these computer-intensive applications is likely to increase even further for two reasons: First, with the popularity of next-generation sequencing methods rising, the number of measurements per sample will soar. Second, the need to assist researchers in answering questions such as "How many groups are in my data?" or "How robust is the identified clustering?" will increase. Cluster number estimation techniques address these types of questions by repeated use of a cluster algorithm with slightly different initializations or data sets, ultimately performing a sensitivity analysis.

In the past, computing speeds doubled approximately every 2 years via increasing clock speeds, giving software a "free ride" to better performance [4]. This is now over, and such automatic performance improvements are no longer possible. As clock speeds are stalling, the increase in computational power is now due to the rapid increase of the number of cores per processor. This makes parallel computation a necessity for the time-consuming analyses in the laboratory. Generally, two parallelization schemes are available. The first is based on a network of computers or computing nodes. The idea of such a master-slave parallelization is to parallelize independent tasks using a network of one master and several slave computers. While there is no possibility for communication between the slaves, this approach best fits scenarios where the same serial algorithm is started several times on different relatively small data sets or different analyses are calculated in parallel on the same data set. Data set size matters here, as distribution of large data sets is time consuming and requires all computers to have the appropriate memory configuration. The second approach called shared memory parallelization is used to parallelize the implementation of an algorithm itself. This is an intrinsic parallelization via different interwoven sub-processes (threads) on a single multi-core computer accessing a common memory, and requires a redesign of the original serial algorithm.

### Master-slave parallelization

Master-slave parallelization is heavily used by computer clusters or supercomputers. The Message Passing Interface (MPI) [5] protocol is the dominant model in high-performance computing. Without shared memory the compute nodes are restricted to process independent tasks. As long as the load-balancing of the compute nodes is well handled, the parallelization of a complex simulation scales linearly with the number of compute nodes. In contrast to massive parallel simulation runs of complex algorithms, master-slave parallelization is also used for parallelizing algorithms. For this task, a large dataset is usually first split into smaller pieces. The subsets are then distributed through a computer network and each compute node solves a subtask for its subset. Finally, all results are transferred back to the master computer, which combines them to a global result. The user interacts with the hardware cluster through the master computer or via a web-interface. However, in addition to hardware requirements, such as minimal amount of memory that are imposed on each compute node, the effort of distributing the data and communicating with nodes of the computer network restricts the speedup achievable with this method. An approach similar to MPI by Kraj et al. [6] uses web-services for parallel distribution of code, which can reduce the effort for administrating a computer cluster, but is platform-dependent. A very popular programming environment in the bioinformatics and biostatistics community is R [7,8]. In recent years several packages (snow, snowfall, nws, multicore) have been developed that enable master-slave parallelized R programs to run on computer cluster platforms or multi-core computers, see Hill et al. [9] for an overview of packages for parallel programming in R.

### Shared memory parallelization

Today most desktop computers and even notebooks provide at least dual-core processors. Compared to master-slave parallelization, developing shared-memory software reduces the overhead of communicating through a network. Despite its performance in parallelizing algorithms, shared memory parallelization is not yet regularly applied during development of scientific software. For instance, shared memory programming with R is currently rather limited to a small number of parallelized functions [9].

Shared-memory programming concepts like the Open Multi-Processing (Open MP) [10] are closely linked to thread programming. A sequential program is decomposed into several tasks, which are then processed as threads. The concept of thread programming is available in many programming languages like C (PThreads or OpenMP threads), Java (JThreads), or Fortran (OpenMP threads) and on many multi-core platforms [11]. Threads are refinements of a process that usually share the same memory and can be separately and simultaneously processed, but can also be used to imitate master-slave parallelization by avoiding access to shared memory [11]. Due to the mostly used shared memory concept, communication between threads is much faster than the communication of processes through sockets. In a multi-core parallelization setting there is no need for network communication, as all threads run on the same computer. On the other hand, as every thread has access to all objects on the heap there is a need for concurrency control [12]. Concurrency control ensures that software can be parallelized without violating data integrity. The most prominent approach for managing concurrent programs is the use of locks [10]. Locking and synchronizing ensures that changes to the states of the data are coordinated, but implementing thread-safe programs using locks can be fatally error-prone [13]. Problems might occur when using too few locks, too many locks, wrong locks, or locks in the wrong order [14]. For instance an implementation may cause deadlocks, where two processes are waiting for each other to first release a resource.

In the following we describe a new multi-core parallel cluster algorithm (McKmeans) that runs in shared memory, and avoids locks for concurrency control. Benchmark results on artificial and real microarray data are shown. The utility of our computer-intensive cluster method is further demonstrated on cluster sensitivity and cluster number estimation of high-dimensional gene expression and SNP data.

## Implementation

### Multicore k-means/k-modes clustering

Clustering is a classical example of unsupervised learning, i.e. learning without a teacher. The term cluster analysis summarizes a collection of methods for generating hypotheses about the structure of the data by solely exploring pairwise distances or similarities in the data space. Clustering is often applied as a first step in data analysis for the creation of initial hypotheses. Let *X *= {**x**_1_, ..., **x**_*N*_} be a set of data points with the feature vector **x**_*i *_∈ ℝ^*d*^. Cluster analysis is used to build a partition of a data set containing *k *clusters such that data points within a cluster are more similar to each other than points from different clusters. A partition *P *(*k*) is a set of clusters {*C*_1_, *C*_2_, ..., *C*_*k*_} with 0 <*k *<*N *and meets the following conditions:

The basic clustering task can be formulated as an optimization problem:

#### Partitional cluster analysis

For a fixed number of groups *k *find that partition *P*(*k*) of a data set *X *out of the set of all possible partitions Φ (*X*, *k*) for which a chosen objective function *f*: Φ (*X*, *k*) → ℝ^+ ^is optimized. For all possible partitions with *k *clusters compute the value of the objective function *f*. The partition with the best value is the set of clusters sought.

This brute force method is computationally infeasible as the cardinality of the set of all possible partitions is huge even for small *k *and *N*. The cardinality of Φ (*X*, *k*) can be computed by the Stirling numbers of the second kind [15]:

Existing algorithms provide different heuristics for this search problem. k-means is probably one of the most popular of these partitional cluster algorithms [16]. The following listing shows the pseudocode for the k-means algorithm:

**Function **k-means

   **Input**: X = {x_1, ..., x_n} (Data to be clustered)

         k (Number of clusters)

   **Output**: C = {c_1, ..., c_k} (Cluster centroids)

         m: X->C (Cluster assignments)

   **Initialize **C (e. g. random selection from X)

   **While **C has changed

      **For **each x_i in X

            m(x_i) = argmin_j distance (x_i, c_j)

      **End**

      **For **each c_j in C

            c_j = centroid ({x_i | m(x_i) = j})

      **End**

   **End**

Given a number *k*, the k-means algorithm splits a data set *X *= **{x**_1 _..., **x**_*n*_} into *k *disjoint clusters.

Hereby, the cluster centroids *μ*_1_, ..., *μ*_*k *_are placed in the center of gravity of the clusters *C*_1_, ..., *C*_*k*_. The algorithm minimizes the objective function:

This amounts to minimizing the sum of squared distances of data points to their respective cluster centroids. k-means is implemented by repeating two major steps, which reassign data points to nearest cluster centroids and update centroids (often also called prototypes) for the newly assembled cluster. A centroid *μ*_*j *_is updated by computing the mean of all points in cluster *C*_*j*_:

#### k-modes clustering for SNP data

Data from SNP profiles can be encoded as a vector of categorical data representing homozygous reference, heterozygous, and homozygous alternative as 0, 1, and 2. For instance, a SNP *s *has two alleles A and T. The three possible genotypes are AA, AT, and TT. A data point *x *is represented as a vector of SNP values. For measuring similarity of two SNP samples, the allele sharing distance (ASD) has been proposed [17]. Recently, it has been shown that ASD provides sufficient information for separating subpopulations using SNPs [18,19]. The allele sharing distance *d*(*x*, *y*) for calculating the distance between data point *x *and *y *is defined as:

where:

To incorporate SNP data, the centroid update step of the k-means algorithm is adapted to calculate centroids from categorical data [20]. Cluster centers are now calculated by counting the frequency of each genotype and using the most frequent genotype (mode) as the new value.

### Parallel k-means/k-modes in shared memory

The k-means/k-modes algorithm is parallelized by simultaneously calculating

(a) the minimum distance partition (first **for **loop in function k-means) and subsequently

(b) the centroid update (second **for **loop).

**Figure 1:**
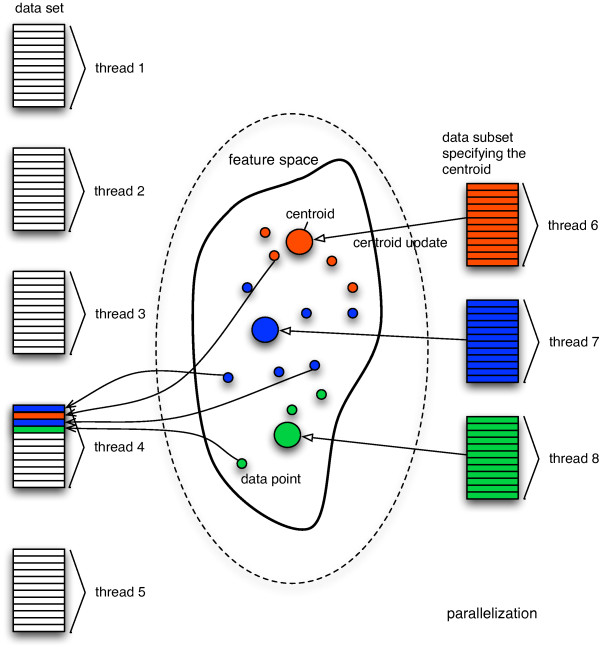
**Basic design of the multicore k-means algorithm**. The data is split and implicitly assigned to different threads (left). Additional threads are used for centroid update (one thread for every centroid). Centroids are initialized randomly. During the cluster assignment step the nearest centroid for each data point is searched and is updated accordingly. Additionally, each data point is written to the list of members of its nearest centroid (right). Simultaneous write access to these lists is possible via software transactional memory.

That means that the complete data set is split into several subsets (Figure 1, left), and nearest centroid search is then performed in an individual thread for that subset, effectively parallelizing the minimum distance search. Simultaneous write access, to the data structures (lists) containing these data points (Figure 1, right), which is not possible in a master-slave scenario, is possible through a transactional memory system (see below). Centroid update is also parallelized by calculating the new location for every centroid from the previously found minimum distance partition (Figure 1, right).

Instead of explicitly controlling thread concurrency, we here use the concept of transactional memory to indirectly guarantee thread safety (e.g. being lock-free). The number of threads used is influenced by two factors: For calculating the minimum distance partition, the number of data threads equals the number of available CPU cores. Furthermore, each centroid is managed by its own thread. This means that during the assignment step, data is continually sent to the centroids from all data threads, and the centroid update is performed with *k *threads in parallel.

#### Transactional memory

In shared memory architectures, there is a need for concurrency control. Simultaneously running threads can process the same data and might also try to change the data in parallel. Opposed to the low-level coding via locking and unlocking individually memory registers, transactional memory provides high-level instructions to simplify writing parallel code [21,22]. The concept of software transactional memory (STM) that we use here is a modern alternative to the lock-based concurrency control mechanism [23,24]. It offers a simple alternative to this concurrency mechanism, as it shifts the often complicated part of explicitly guaranteeing correct synchronization to a software system [25]. The basic functionality of software transactional memory is analogous to controlling simultaneous access via transactions in database management systems [26]. Transactions monitor read and write access to shared memory and check whether an action will cause data races. The STM system prevents conflicting data changes by rolling back one of the transactions. Transactions ensure that all actions on the data are atomic, consistent, and isolated. The term atomic means that either all changes of a transaction to the data occur or none of them does. Consistent means that the new data from the transaction is checked for consistency before it is committed. Isolated means that every transaction is encapsulated and cannot see the effects of any other transaction while it is running. As a consequence, transactional references to mutable data via STM enables sharing changing state between threads in a synchronous and coordinated manner.

**Figure 2:**
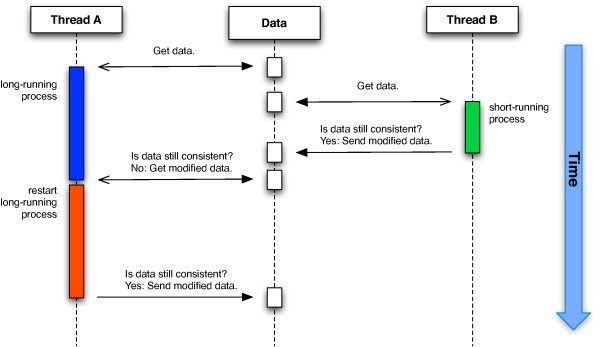
**Software transactional memory**. Software transactional memory circumvents the need for explicit locking of resources. All changes to the state of data are encapsulated in transactions, i.e. every thread has a copy of its working data and can change its value. During submission of the changes to the shared memory, the consistency of the internal state is checked. If no interim changes occurred, the submission is performed. If another thread working on another copy of the same data has meanwhile submitted its changes, the transaction is rejected and restarted with a new copy of the data.

Implementations of software transactional memory can be divided into two categories called direct-update and deferred-update STMs [25,27]. In our implementation, we use a deferred-update STM (see Figure 2). Transactions in deferred-update STM systems obtain a copy of the original data and process their changes. Before committing the changes to the shared memory, conflicts are checked by the STM system, and conflicting transactions are rejected. As side effects from conflicting transactions do not affect the shared memory, there is no need for restoring a consistent memory state. Threads concurrently execute all of their modifications to the shared data without locking other threads. However, before committing the changes, the system checks whether other threads have altered the data in use. If so, the transaction is retried until a consistent commit can be performed. Through the use of atomic blocks encapsulating code fragments, parallel code can be implicitly defined without knowledge about locking strategies or thread handling. The STM system guarantees to handle the atomic block correctly.

### Cluster number estimation

Cluster number estimation can be linked to an assessment of the stability of the clustering. This issue is often discussed in cluster analysis, as the number of clusters in the data is usually unknown [28-30]. It has been shown that a repeated cluster analysis with different methods, parameters (especially a different number of assumed clusters), feature sets, or sample sizes can help to reveal the underlying data structure. For instance, the bootstrap technique can be used for estimating the number of clusters [31]. If the fluctuations among the partitions are small compared to random clustering, the clustering is called robust, and that particular model is chosen. Although there are few theoretical findings on the stability property of clusterings, this methodology has proven to work well in practice [32-34]. For stability evaluation of repeated clusterings, methods that measure the similarity of a clustering relative to some instance are used. These methods measure different characteristics of the identified partitions or sequences of partitions, thus implying repeated calculations. They can be subdivided into three groups [15]:

1. Internal criteria: Measure the overlap between cluster structure and information inherent in the data, for example silhouette, inter-cluster similarity.

2. External criteria: Compare different partitions, for example Rand index, Jaccard index, Fowlkes and Mallows.

3. Relative criteria: Decide which of two structures is better in some sense, for example quantifying the difference between single-linkage or complete-linkage.

To demonstrate the quality of cluster algorithms, they are often applied to a-priori labeled data sets and evaluated by an external criterion [28,35]. An external index describes to which degree two partitions agree, given a set of *N *objects *X *= {*x*_1_, ..., *x*_*N*_} and two different partitions *P *= {*C*_1_, ..., *C*_*r*_} and *Q *= {*D*_1_, ..., *D*_*s*_} into *r *and *s *clusters respectively.

#### MCA cluster similarity index

**Figure 3:**
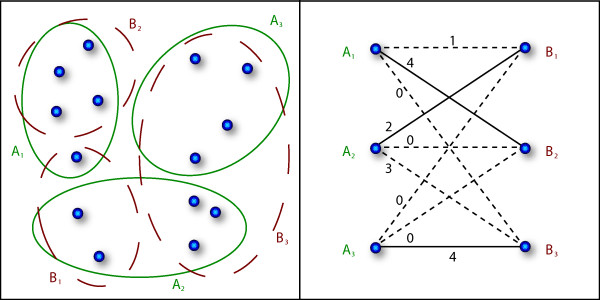
**Example for cluster evaluation via the MCA index**. On the left, two possible partitionings of the data set are shown, i. e. *P *= {*A*_1_, *A*_2_, *A*_3_} and *Q *= {*B*_1_, *B*_2_, *B*_3_}. The bipartite matching graph is given on the right. Each edge is annotated with the number of intersecting data points in both partitionings. The solid lines mark the maximal matching edges. In this example the MCA index is  = 0.71.

For the evaluation of the experiments, we here use a measure that is based on the pairwise similarity between set partitions and can be interpreted as the mean proportion of samples being consistent over the different clusterings [32,33]. Because this index behaves linearly in the number of data points it offers a better interpretability in terms of proportion of samples moving between clusters. There is no such intuitive interpretability for quadratic validity measures like Rand or Jaccard index [36,37]. The concept is illustrated in Figure 3. In the left part of Figure 3 two partitionings *P *and *Q *are compared. The correspondence or similarity  between two clusters *C*_*i *_and *D*_*j *_is given by the size of the intersection set |*C*_*i *_∩ *D*_*j*_|. The idea of the maximum cluster assignment (MCA) index is to find a bijective mapping *π*: {1 ... *k*} → {1 ... *k*} that maps each cluster from one clustering *P *to its corresponding cluster in *Q *such a way that the sum over all similarities  is maximized. The bold lines in the right part of Figure 3 mark the maximum matching nodes in the bipartite graph representation. In this example, the best mapping is *A*1 ↔ *B*2, *A*2 ↔ *B*1, *A*3 ↔ *B*3. The MCA index is then defined as the ratio of the number of data points in the intersection sets of the corresponding clusters to the overall number of data points:

The normalization factor  bounds the index into (0, 1], where a value of 1 denotes a perfect matching between the two clusterings, i.e. the two partitions are identical up to a permutation of their components. The remaining problem is to find the best mapping *π*(·). This is a well-known problem in discrete mathematics called linear assignment problem (LAP [38]). In the current implementation, we use the algorithm by Jonker & Volgenant (1987) [39] that runs in (*k*^3^) after building the assignment matrix, which can be done in (*n*).

#### Correction for chance

Cluster validity indices are used to quantify findings about results of a cluster analysis. They do not include information about a threshold for distinguishing between high and low values. Statistical hypothesis testing provides a framework to distinguish between expected and unusual results based on a distribution of the validity index [40,41]. The null hypothesis is chosen to reflect the case of no inherent cluster structure.

Different null hypotheses, which lead to different expected values of a cluster validation index and all reflect a specific context of the clustering can be designed. Yet, due to the complex characteristics of the baseline distributions and the validation indices it is often not possible to deduce a formula for the expected value of a corrected index [15]. In this case, a Monte Carlo analysis can assist to reveal the distribution of these indices under the chosen null hypothesis. In a Monte Carlo simulation, several independent test sets are sampled from a given baseline distribution. The chosen validity index is then evaluated for the random data sets. This gives an estimate for the expected value of a validity index under an empirical baseline distribution. Then, the baseline distribution is used to correct the validation index *I *for randomness:

where *I*_*max *_is the maximum value (which is 1 in case of MCA-index) and *E*(*I*) is the expected value under a random hypothesis.

**Figure 4:**
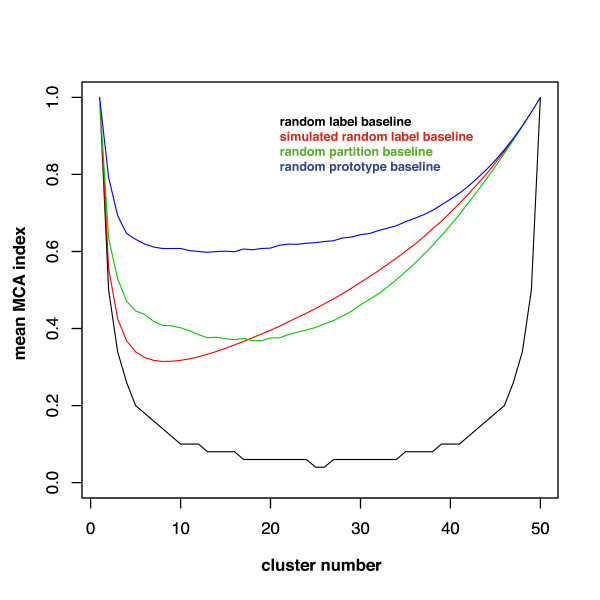
**Comparing different baseline distributions for clustering**. Baselines for clustering an artificial data set containing 50 one-dimensional points. For each partitioning into *k *= {1, ..., 50} clusters, the average value of the MCA index from 500 runs is plotted. The different baselines are from bottom to top: black = random label, red = simulated random label, green = random partition, blue = random prototype. It can be seen that the random label baseline is a lower bound for the MCA index, whereas the simulated random label and random partition baselines are much tighter. The data-driven random prototype baseline builds the tightest bound for the MCA index.

In the following, we consider three baseline scenarios that we call the random label hypothesis, the random partition hypothesis, and the random prototype hypothesis (see Figure 4).

#### Random label hypothesis

The random label hypothesis simulates the worst case behavior of a clustering. Each data point is randomly assigned to one of the *k *clusters such that no cluster remains empty, i.e. ∀*x*_*i *_∈ *X *assign *x*_*i *_to cluster *C*^*r*^, with *r *uniformly chosen from {1, ..., *k*} and all *C*^*r *^≠ ∅. The Monte Carlo simulation for the empirically expected value of the MCA index under this baseline is shown in Figure 4. For the MCA index, the expected value under this hypothesis can also be derived analytically:

• If  is an integral number, the expected value of matching points between partitions is .

• Otherwise, there is at least one cluster expected to have more matching data points, i.e. the expected value is .

• *E*(*M CA*) is not monotonically decreasing with *n*, but has a minimum at , see Figure 4.

The expected value of the MCA index is:

The number of matching points between partitions cannot decrease when choosing another baseline hypothesis, i.e. this hypothesis reflects the lower bound of the MCA index. Due to the limitations of the Monte Carlo simulation, the expected value of the simulated random label baseline stays constantly above the theoretical limit unless *n *≫ *k *(Figure 4).

#### Random partition hypothesis

The random partition hypothesis simulates the general behavior of randomly clustering a data set. Under this hypothesis, every partition of *n *data points into *k *clusters is assumed to be equally probable. The number of possible partitions is given by the Stirling numbers of the second kind [42]: . Even for small *n *and *k *an exhaustive computation of all possible partitions is not feasible. To give an estimate of the expected value under this hypothesis, a Monte Carlo simulation can be used (Figure 4).

#### Random prototype hypothesis

In contrast to the previous hypotheses, the random prototype hypothesis simulates the average behavior of a clustering with respect to a given data set. *k *cluster prototypes *c*_*j *_are chosen randomly, and according to these prototypes, an assignment is performed, e.g. the nearest neighbor rule: ∀ *x*_*i *_∈ *X *assign *x*_*i *_to cluster *C*^*r *^if *r *= *argmin*_*j*_||*x*_*i *_- *c*_*j*_||^2^. Varying the assignment rule enables the simulation of different cluster algorithms (here: nearest centroid for k-means type clustering). Under this hypothesis, the generated partitions are data-driven and best reflect the random baseline clustering for each data set (Figure 4).

### Choosing the appropriate clustering

With a fast cluster number estimation, a two step procedure can be executed to choose the appropriate clustering. The first step consists of choosing a set of *k*'s that have the highest robustness. For this task we and others propose the sensitivity of the clustering as a measure, see the preceding section [32-35,43-47]. Robustness analysis is based on the observation that for a fixed number of clusters, repeated runs of a cluster algorithm on a resampled dataset often generate different partitions. The robustness of k-means is also affected by different random initializations. To reduce this effect, k-means is restarted repeatedly for each resampled dataset. Only the result with minimal quantization error is then included into the list of generated partitions. In this regard, the median value of the MCA index from comparing all generated partitions to another can serve as a predictor for the correct number of clusters. We define the best number of clusters *k *as the one with maximal distance between median MCA index from cluster results and median MCA index from the random prototype baseline. Statistical hypothesis testing (e.g. Mann-Whitney-test) can be used to rate the significance of the observed clusterings with respect to the baseline clustering and thus can serve to reject a clustering altogether, meaning no structure in data can be found.

In the second step, we choose the partition with the smallest quantization error for the selected *k*'s. As k-means does not guarantee to reach a global optimum, but convergence to a local optimum is always given [48], we use the strategy of restarts with different initializations [15]. Finally, the result with the minimal quantization error (least mean squared error) is selected as the best solution. For extremely large data sets, this strategy requires a fast implementation, as several hundreds of repetitions may be necessary [20].

## Results

To illustrate the utility of our multi-core parallel k-means algorithm we performed simulations on artificial data, gene expression profiles and SNP data. All simulations of McKmeans were performed on a Dell Precision T7400 with dual quad-core Intel Xeon 3.2 GHz and 32 GB RAM. The four cores on each CPU share 6 MB of cache. Simulations were partly compared to two reference implementations, namely the single-core k-means function implemented in R [7] and the network-based ParaKMeans [6] algorithm. For the k-means function from R (version 2.9), simulations were also performed on the Dell T7400.

ParaKMeans was tested on the web interface at http://bioanalysis.genomics.mcg.edu/parakmeans. Some of our larger test data could not be processed due to either a slow data loading routine (R) or memory limitations on the master computer. These runtime performance comparisons between different implementations (languages, hardware, software paradigms) can only illustrate a rough difference between single and multi-core algorithms and should not be regarded as benchmarks.

### Artificial data

#### Artificial data sets without cluster structure

**Figure 5:**
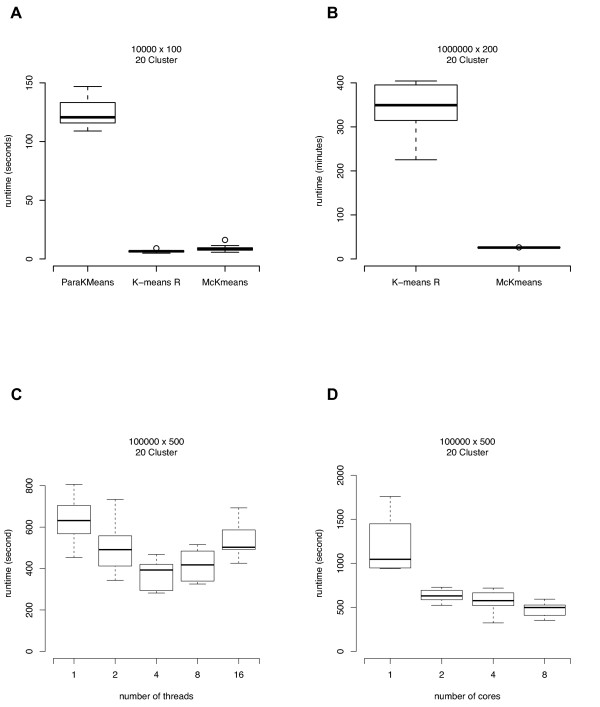
**Runtime performance of ParaKMeans, k-means R, and McKmeans on the artificial data sets**. Benchmark results for the simulated data sets (no cluster structure imposed, features chosen uniformly from [0, 1]) comparing the runtime of ParaKMeans, k-means R, and McKmeans. For the smaller data set (panel A) the computational overhead of the parallelization negatively affects the runtime. For the larger data set (1 million cases, panel B) an improvement of the runtime by a factor of 10 can be observed. The network-based parallelization algorithm ParaKMeans is significantly slower than McKmeans. Panel C shows the dependency of the runtime on the number of threads used (Kruskal-Wallis test: *p *= 1.15 × 10^-5^) and Panel D the number of cores used (Kruskal-Wallis test: *p *= 4.59 × 10^-6^) for a data set of 100000 cases and 500 features. Each box summarizes the results from 10 repeated clusterings (median and interquartile range).

We generated data sets without imposing a cluster structure. As the k-means algorithm is guaranteed to converge to a clustering, the median runtime of the algorithm on such data sets was used as a performance measure. We generated three simulated data sets (10000 samples with 100 features, 100000 samples with 500 features, 1000000 samples with 200 features). Each feature is uniformly distributed over the interval [0,1] to minimize the effect of random initializations. The performance of clustering the data sets into 20 clusters is summarized in Figure 5A. Each box summarizes the results of 10 repeated clusterings (median and interquartile range). In case of the small data set the computational overhead of the thread management negatively affects the runtime. For the extremely large data set, an improvement of the runtime by a factor of 10 can be observed (Figure 5B).

The influence of changing the number of threads (1, 2, 4, 8, 16) for calculating the minimum distance partition (the number of threads used for the centroid assignment is always k) in McKmeans is shown in Figure 5C. Each box summarizes the results of 10 repeated clusterings for a data set (100000 samples with 500 features). The choice of the number of threads shows best performance if it is in the range of the number of physical CPUs to the number of cores, i.e. 2 to 8 cores.

We also performed a cluster analysis with McKmeans for different numbers of computer cores on a data set (100000 samples with 500 features). A summary of the experiments using 1, 2, 4, and 8 cores is shown in Figure 5D. Using 4 cores resulted in a runtime improvement by a factor of 2 compared to the single-core experiment. With 8 cores, the CPU usage rate never exceeded 600%, i.e. not all cores were used during calculations.

#### Artificial data sets with gene cluster structure

We simulated clustered data sets using multivariate normal distributions as the basis for each cluster [49]. An artificial microarray experiment consists of *n *microarrays being composed of *p *genes. An experiment is sampled to contain exactly *k *gene clusters. Within-cluster variance and between-cluster variance are used to assemble a set of exactly *k *well-formed gene clusters as follows: At first, *k *pair-wise equidistant gene cluster centroids *μ*_*k *_are drawn from an interval around 0 with the variance set to the between-cluster variance . Each gene is assigned to one of the *k *gene cluster centroids. Then, a gene-specific mean *μ*_*g *_is drawn from a normal distribution with the mean set to the assigned cluster centroid *μ*_*k *_and variance set to the within-cluster variance . The variance of an individual gene over *n *microarrays  follows a *χ*^2 ^distribution with *n *degrees of freedom. To get an unbiased estimate of the variance, it is divided by *n *- 1, i.e. , with *x *~χ^2 ^[50]. Only a small fraction of genes in the same cluster is set to have a non-zero correlation. The probability of any gene-pair to be correlated is set to *c *= 5 * 10^-(*log*(*p*)+2)^. For each cluster the number of correlated genes is determined by a Poisson distribution with mean equal to , where *p*_*k *_is the number of genes in cluster *k*. If gene *g*_*i *_and *g*_*j *_are correlated, the covariance is calculated from a product of , and the correlation *r *is drawn randomly from a uniform distribution (*r *~*U *(-1, 1)) [6]. The covariance matrix Σ and the gene mean vector *μ*_*g *_are then used to simulate the different artificial microarrays. An artificial microarray is calculated from Σ and *μ*_*g *_using the triangular factorization method. A matrix Σ can be factored into a lower triangular matrix *T *and its transpose *T'*, Σ = *TT'*. It follows that *X *= *YT *+ *μ*_*g *_~*N*_*k*_(*μ*_*g*_, Σ), with a matrix *Y *~*N*_*k*_(0, *I*). The factorization is done with the Cholesky decomposition of Σ [49].

**Figure 6:**
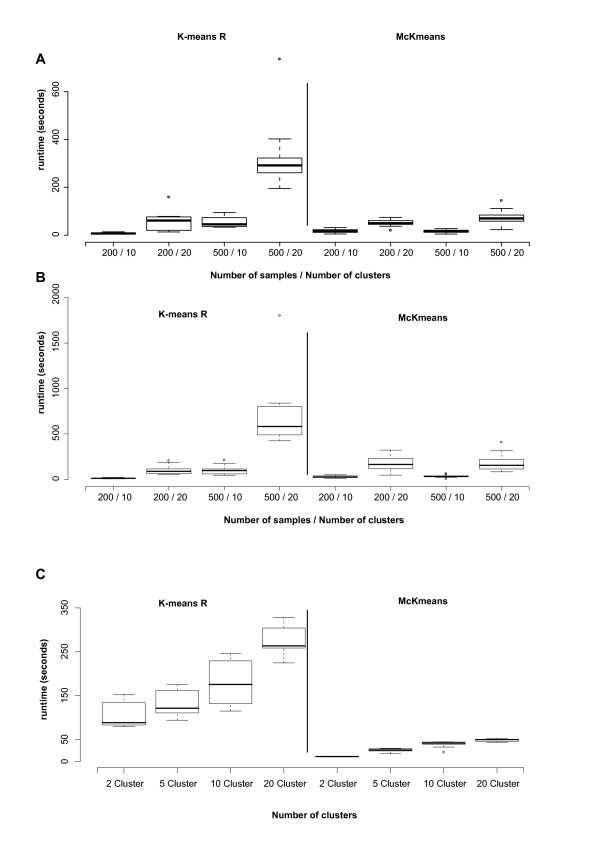
**Runtime performance of R and McKmeans on the microarray data**. Benchmark results for the artificial microarray data set with 50000 (panel A) and 100000 (panel B) genes, 200/500 arrays, and 10/20 clusters comparing the runtime of k-means R, and McKmeans. Each box summarizes the results from 10 repeated clusterings. Panel C shows the runtime for clustering genes (22277) from 465 cell lines of Smirnov et al. [51]. The parallelization leads to a runtime improvement by a factor of 10.

We generated artificial microarray experiments with different number of genes, arrays, and clusters (p = 50000, 100000, n = 200, 500, k = 10, 20). Benchmark results for these data sets are given in Figures 6A+B. Each box summarizes the results of 10 repeated clusterings. Both McKmeans and k-means R use the Mersenne Twister to generate random numbers. When started with the same seed value, our implementation of k-means reproduces exactly the same results as computed by the reference implementation in R.

#### Cluster number estimation on artificial data

To further illustrate the need for a high computational speed of cluster algorithms, we performed simulations to infer the number of clusters inherent in a data set. The stability is measured by comparing the agreement between the different results of running k-means on subsets of the data. The agreement is measured with the MCA index, and correction for chance is done using the random prototype hypothesis. Here, we simulated the clustered data set using separate multivariate normal distributions as the basis for each cluster. We generated a data set with 100000 cases containing 3 clusters in 100 dimensions. The data set was resampled 10 times leaving out  data points. The effect of resampling on the stability of the clustering can be reproduced on this data. The experiment correctly predicts a most stable clustering into 3 clusters. Total running time was 204.27 min. In the simulation 380 separate clusterings were performed. We also performed a cluster number estimation for every artificial data set mentioned in this paper. All simulations predicted the correct number of clusters, see supplementary material (Additional file 1).

### Gene expression profiles

#### Smirnov microarray data

We also compared the algorithms on gene expression profiles from Smirnov et al. [51] with 22277 genes and 465 cell lines. They used data from cells collected at baseline and 2 and 6 h after exposure to 10 Gy of ionizing radiation. We performed two experiments on this data, one comparing runtimes of clustering genes and a second one performing a cluster number estimation for grouping cell lines. Results of the runtime experiments are given in Figure 6C. Here, each box summarizes the results from 10 repeated clusterings. Our multi-core algorithm performs up to 10 times faster than the single-core k-means algorithm included in R. In the cluster number estimation experiment, the objective was to find the best clustering of the 465 profiles using all available 22277 genes. We performed 3800 cluster runs (*k *= 2 ... 20, 100 repetitions each for the clustering and the random prototype). The best clustering was found with *k *= 4 clusters. These four clusters do not correspond to the quantitative phenotypes induced by radiation exposure (see Table 1). This suggests and supports the findings of Smirnov and co-workers that indicate a highly individual response to the damage at the expression level, and not a uniform mechanism of how cells deal with this radiation exposure.

**Table 1:** Clustering of gene expression profiles from Smirnov et al. [51].

	*C*^1^	*C*^2^	*C*^3^	*C*^4^
0 hr	67	21	43	24

2 hrs	68	22	37	28

6 hrs	62	36	31	26

Cluster results for the best clustering with four clusters. Each entry shows the number of profiles that are in Cluster *C*^*i *^and one of the groups (0 hrs of radiation, 2 hrs of radiation, and 6 hrs of radiation).

### Single nucleotide polymorphism data

#### HapMap SNP data

For evaluating the performance of McKmeans in clustering SNP data, we used the HapMap Phase I SNP dataset [52]. The HapMap project collected SNPs from 270 individuals from four ethnic populations: Yoruba in Ibadan, Nigeria (YRI), CEPH (Utah residents with ancestry from northern and western Europe, CEU), Han Chinese in Beijing, China (CHB), and Japanese in Tokyo, Japan (JPT). For the cluster analysis we only used unrelated individuals. The number of unrelated individuals per group is: 60 YRI, 60 CEU, 45 CHB, and 45 JPT. Only SNPs with a minor allele frequency greater than 0.1 have been included, which resulted in 116678 SNPs.

**Figure 7:**
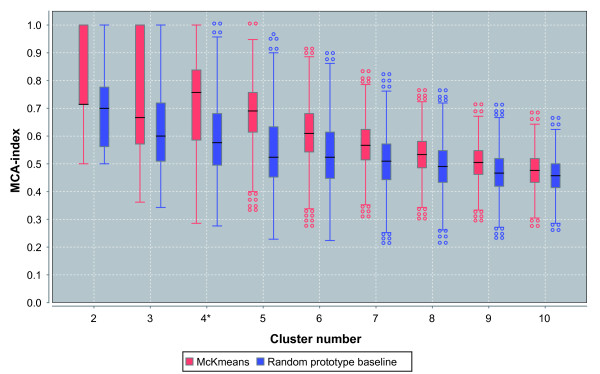
**Clustering and stability estimation for HapMap SNP profiles**. Cluster number estimation via repeated clustering of profiles/subjects for the HapMap data (210 profiles, 116678 SNPs). For each *k *∈ {2, 3, ..., 10}, 1000 repeated cluster runs were performed. For each cluster number, two boxplots are shown, one summarizing the MCA values from the pairwise comparisons of all cluster results (left), and the other one for the results from the random prototype baseline (right). A higher value indicates increased stability.

We performed a cluster number estimation for this data (number of rows 210 (profiles), number of columns 116678 (SNPs)). For each *k *{∈ 2, 3, ..., 10}, we performed 1000 runs of clustering on a resampled data set of row size 195 (jackknife ). The results are illustrated in Figure 7. For each *k*, two boxplots are shown, one summarizing the MCA values from the pairwise comparisons of all cluster results and the other one showing the results of the random baseline. The best clustering (maximal distance between medians) is reported for *k *= 4 (Mann-Whitney test: *p *< 1.0 × 10^-16^). We then computed 1000 repeated runs of k-means with *k *= 4. The clustering with the minimal quantization error is given in Table 2. The reported clustering essentially coincides with the different populations. All individuals from CEU form a cluster, as well as individuals from YRI do. One individual from CHB is clustered into the group of JPT, and 3 individuals from JPT are clustered into the group of CHB. This gives an overall accuracy of 98.1% for separating the population by clustering the available SNP data.

**Table 2:** Clustering of SNP profiles from the HapMap data set. Cluster results for the best clustering with four clusters. Each entry shows the number of individuals that are in Cluster *C*^*i *^and one of the populations (CHB, JPT, CEU, YRI).

	*C*^1^	*C*^2^	*C*^3^	*C*^4^
CHB	44	1	0	0

JPT	3	42	0	0

CEU	0	0	60	0

YRI	0	0	0	60

## Discussion and Conclusion

Fast algorithms are increasingly becoming important in the individual laboratory, as the sizes of data sets grow and computational demands rise. We have devised a variant of the popular k-means/k-modes algorithm that effectively utilizes current multi-core hardware, so that even complex cluster number estimations for large data sets are possible on a single computer. Computer-intensive bioinformatics software is frequently parallelized using a network-based strategy. Such a parallelization can be very efficient when the same algorithm has to be started several times on different data sets of moderate size, or when different analyses have to be calculated in parallel on the same data set. However, this approach also requires additional effort and equipment, like specialized hardware for fast communication between computers, multiple software installations in heterogeneous environments, or compute cluster administration. For multi-core parallelization, OpenMP and functional programming languages provide a basic parallelization scheme through the parallel execution of loops. More efficient parallelization can be achieved through direct use of threads and locking variables, which requires additional effort for concurrency control as well. We have designed a highly efficient parallel k-means algorithm that utilizes transactional memory, guarantees concurrency, and can serve as a template for other parallel implementations. We achieve a performance increase that scales well with the available resources. An even more dramatic performance gain could be measured in the comparison to the single core k-means implementation: On 8 cores, the parallel implementation attained a 13-fold speed increase (338 min vs 25 min) for a large data set of 1 million cases with 200 dimensions. This disproportionately high increase is partly due to different data loading times of the R implementation and our Java application. For smaller data sets, the highly efficient R implementation, which calls compiled C code, outperforms both our multi-core implementation and a network-based reference implementation on singular cluster runs. Cluster number estimation is often discussed in conjunction with cluster analysis methods, as the number of clusters is an unknown prior [28,30]. For instance, the gap statistic can be used to search for a strong change in within-cluster dispersion across different numbers of clusters [53]. Such approaches that are based on internal cluster measures favor highly compact clusters. Other methods relying on resampling strategies combined with external cluster evaluation measures have been used to additionally incorporate the stability of single clusters [31-33]. Consensus clustering assesses the stability as the percentage of object pairs clustered together [29,54,55]. Here, the consensus matrix scales quadratically with the number of objects, and therefore becomes infeasible in clustering extremely large data sets. In contrast, our cluster number estimation method based on resampling and a similarity measure that is linear in the number of objects provides an easy interpretation of the results: Instead of considering pairs of objects, we calculate the percentage of objects clustered together across multiple clusterings. For cluster number estimation across repeated runs of an algorithm, fast implementations become even more important, as simulation time can be extensive even for small data sets. For instance, for a cluster number estimation on a data set consisting of 100 cases and 5000 gene expression values, our multi-core algorithm reduces the runtime from 467 min (using the R implementation) to 115 min. For larger data, cluster number estimation now becomes feasible and can give new insights into the data, like suggesting a highly individual radiation-induced response of B cells at the expression level (Smirnov data), or showing that a grouping of individuals is actually possible on the basis of single nucleotide polymorphisms (HapMap data). Our evaluations of the McKmeans algorithm show that it is fast, achieves the same accuracy as the single-core reference implementations, and is able to cluster extremely large data sets. Furthermore, the Java implementation is easily deployable on different hardware and software platforms. It runs on a single desktop computer and is able to perform complex cluster number estimation experiments due to parallelization.

## Availability and requirements

**Figure 8:**
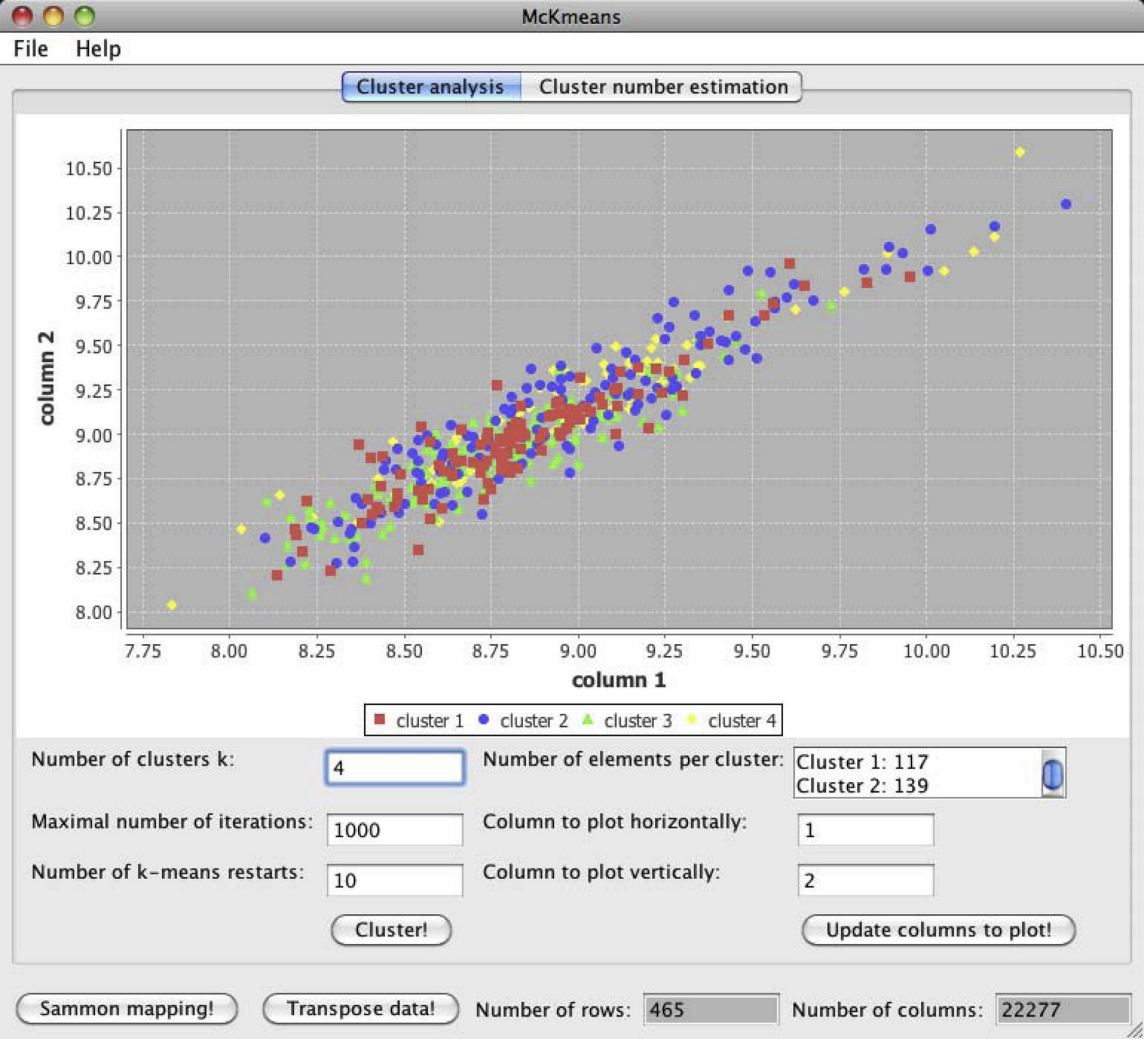
**Graphical user interface**. The GUI supports clustering of microarray (real valued) and SNP data. Clustering subjects and clustering genes/SNPs can be done by transposing the imported data set. Gene data can be visualized as a scatterplot from two selected dimensions and via a Sammon mapping. Cluster number estimations can also be visualized (see Figure 7).

The Java software McKmeans supports multi-core and single-core k-means clustering of real valued data, k-modes clustering of SNP data, and for both data types cluster number estimation. There are three possible modes of using McKmeans, the graphical user interface (see Figure 8), the command line version, and the R-package (rMcKmeans).

### Graphical user interface

The graphical user interface (GUI) software is available for download from our website. Usage of the software is described in the built-in help system. The GUI supports clustering of microarray (real valued) and SNP data. Clustering subjects and clustering genes/SNPs can be done by transposing the imported data set. Gene data is visualized as a scatterplot from two selected dimensions. Furthermore, Sammon's projection method can be performed to show a nonlinear two-dimensional projection of the data [56,57]. Results from the cluster number estimation are given as boxplots. We integrated a statistical test (Mann-Whitney test) for computing the significance of the best cluster result. The best clustering and the results of the cluster number estimation can be saved for further analysis with statistical software such as R. All plots can be saved as SVG files.

### Command line usage

For running batch analyses, McKmeans offers a command line interface to all functions of the GUI version. The command line usage is described on our website.

### R package

With the R package "rMcKmeans", the multi-core k-means algorithm is fully integrated into the R software framework. rMcKmeans is based on the Java implementation and is freed from three important limitations in R: (a) R can only process data sets with up to 2 billion entries, while Java supports datasets of size 2^31 ^× 2^31^, (b) loading time of extremely large datasets in R is extensive, and (c) R does not yet support true multi-core programs. All available multi-core packages in R cannot share memory and internally have to replicate the data for every used core, resulting in a lower amount of total memory available for computing.

McKmeans is a Java program implemented in Clojure (freely available at http://www.clojure.org). McKmeans can also be called from R (package rMcKmeans).

• Project name: McKmeans

• Project home page: http://www.informatik.uni-ulm.de/ni/mitarbeiter/HKestler/parallelkmeans

• Operating system(s): Platform independent

• Programming language: Java, R

• Other requirements: Java 1.6 or higher

• License: Artistic License 2.0

• Any restrictions to use by non-academics: no

## Authors' contributions

JMK and HAK designed the study and wrote the manuscript. JMK implemented the algorithm and performed the experiments. All authors read and approved the final manuscript.

## Supplementary Material

Additional file 1 **Additional cluster number estimation results**. Cluster number estimation results are given for random data sets with and without cluster structure. Click here for file
