# Supplementary material for: A highly efficient multi-core algorithm for clustering extremely large datasets
Source: BMC Bioinformatics. 2010 Apr 6;11:169. doi: 10.1186/1471-2105-11-169 (PMC2865495; doi:10.1186/1471-2105-11-169)
Supplement: Additional file 1 — Additional cluster number estimation results. Cluster number estimation results are given for random data sets with and without cluster structure. [file 1471-2105-11-169-S1.PDF]

Supplementary Information: A highly efficient multi-core algorithm for clustering extremely large datasets

Johann M. Kraus and Hans A. Kestler

Results of cluster number estimation

Cluster number estimation can be linked to an assessment of the stability / reliability of the clustering / partition. It has been shown that a repeated cluster analysis with different methods, parameters (especially a different number of assumed clusters), feature sets or sample sizes can help to reveal the underlying data structure. If the fluctuations among the partitions are small compared to random clustering, the clustering is called robust (or stable) and that particular model is chosen. The stability is measured by comparing the agreement between the different results of running McKmeans on subsets of the original data. Results are shown in the following figures for random data sets without and with cluster structure. The agreement is measured with the MCA index. Correction for chance using the random prototype hypothesis is indicated by the blue bar, cluster results are indicated by the red bar. A higher value shows increased stability. The data sets were resampled 10 times leaving out $\sqrt{n}$ data points.

Artificial data sets without cluster structure

We generated three simulated data sets (10000 samples with 100 features, 100000 samples with 500 features, 1000000 samples with 200 features). Each feature is uniformly distributed over the interval [0,1] to minimize the effect of random initializations. Results for the cluster number estimation for $k = 1 \ldots 10$ are shown in the following three figures. Each box summarizes the MCA-index resulting from 10 repeated clusterings (median and interquartile range). Red boxes show results from the McKmeans cluster algorithm, blue boxes show results from the random prototype baseline. For all data sets there is no difference between cluster results and random baseline results confirming the hypothesis of no cluster structure inherent in the data sets.

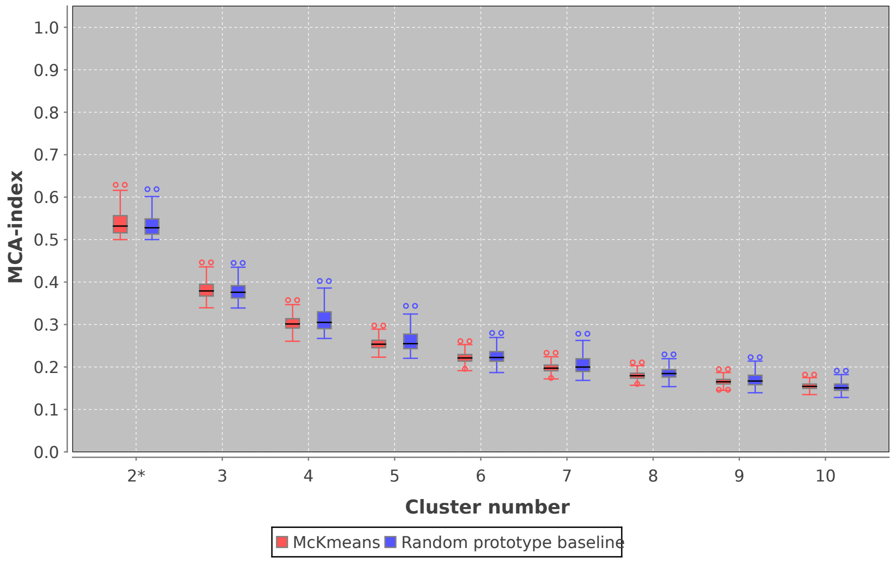

Figure 1: A randomly generated data set (n=10000, d=100) is resampled 10 times and clustered assuming different number of clusters k. Stability is measured over different numbers of clusters with the MCA index and the random prototype hypothesis. Cluster results are shown as red boxes, baseline results as blue boxes. The cluster number estimation method reports no difference between median of cluster results and median of baseline results, i.e. there is no inherent clustering in the data. In fact each sample was drawn from a uniform distribution.

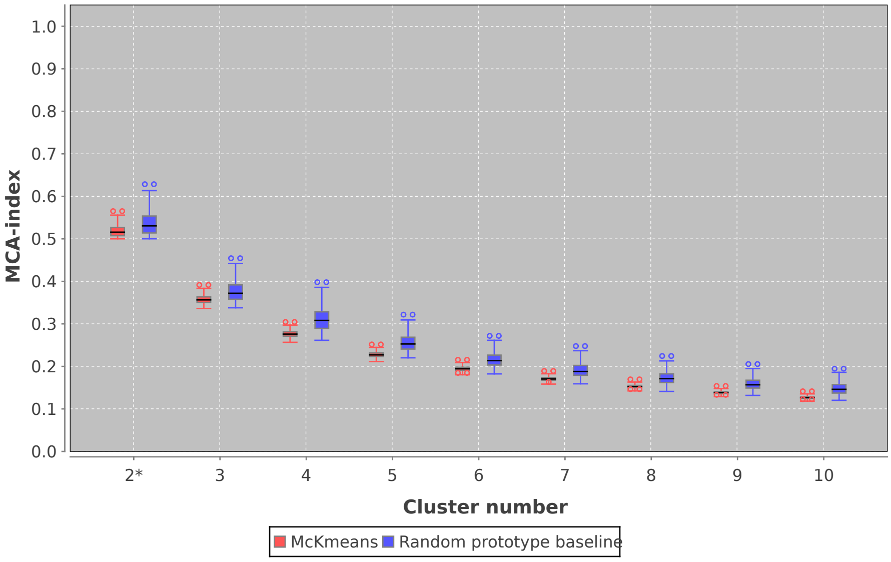

Figure 2: A randomly generated data set (n=100000, d=500) is resampled 10 times and clustered assuming different number of clusters k. Stability is measured over different numbers of clusters with the MCA index and the random prototype hypothesis. Cluster results are shown as red boxes, baseline results as blue boxes. The cluster number estimation method reports no difference between median of cluster results and median of baseline results, i.e. there is no inherent clustering in the data. In fact each sample was drawn from a uniform distribution.

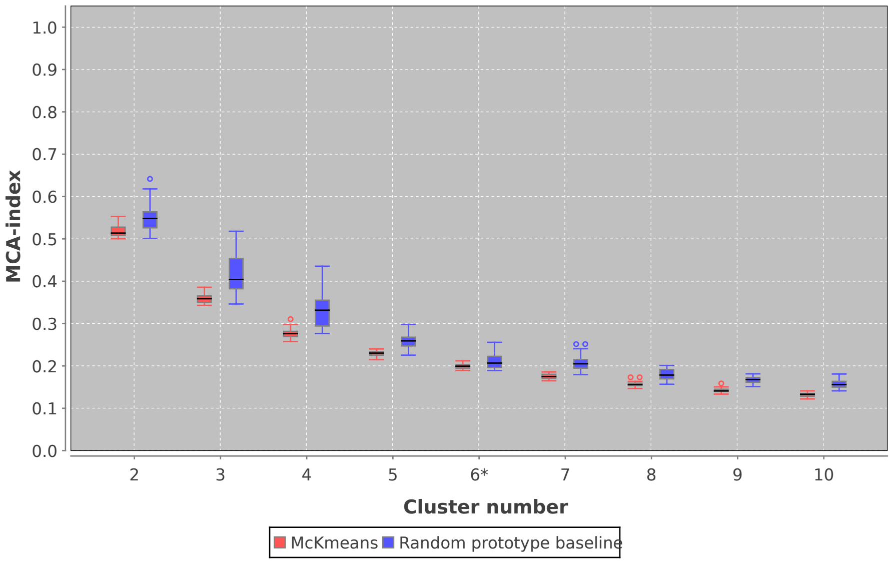

Figure 3: A randomly generated data set (n=1000000, d=200) is resampled 10 times and clustered assuming different number of clusters k. Stability is measured over different numbers of clusters with the MCA index and the random prototype hypothesis. Cluster results are shown as red boxes, baseline results as blue boxes. The cluster number estimation method reports no difference between median of cluster results and median of baseline results, i.e. there is no inherent clustering in the data. In fact each sample was drawn from a uniform distribution.

Artificial data sets with cluster structure

Here, we simulated clustered data sets using separate multivariate normal distributions as the basis for each cluster. We generated several data sets with different number of genes, arrays, and clusters (p = 50000, 100000, n = 200, 500, k = 10, 20). Results for the cluster number estimation for $k = 1 \ldots 10$ (or $k = 1 \ldots 20$, respectively) are shown in the following eight figures. Each box summarizes the MCA-index resulting from 10 repeated clusterings (median and interquartile range). Red boxes show results from the McKmeans cluster algorithm, blue boxes show results from the random prototype baseline. The cluster number estimation method shows the correct predictions for the number of k clusters on all data sets.

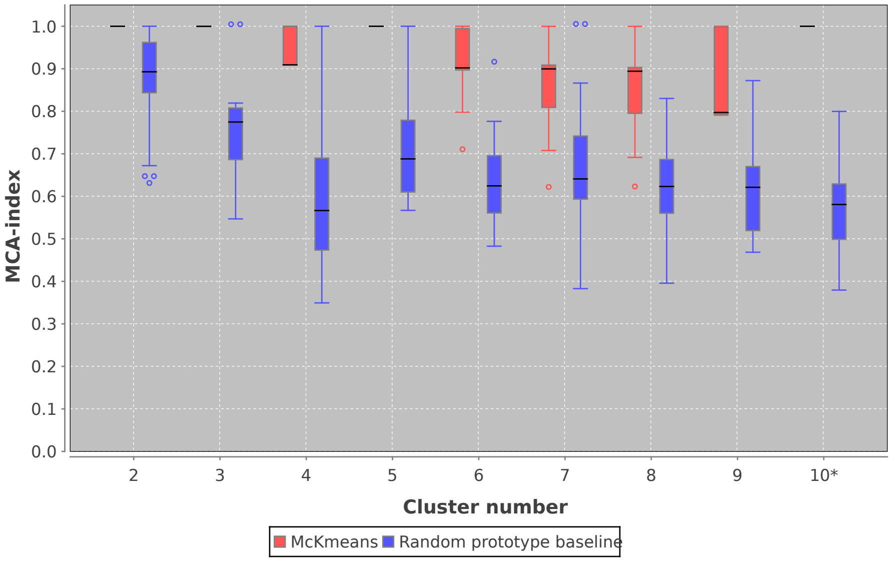

Figure 4: A randomly generated data set (n=50000, d=200, k=10) is resampled 10 times and clustered assuming different number of clusters k. Stability is measured over different numbers of clusters with the MCA index and the random prototype hypothesis. Cluster results are shown as red boxes, baseline results as blue boxes. The best stability (greatest difference between median of cluster results and median of random baseline) is reported for $k = 10$, which is the number of clusters the data set was generated with.

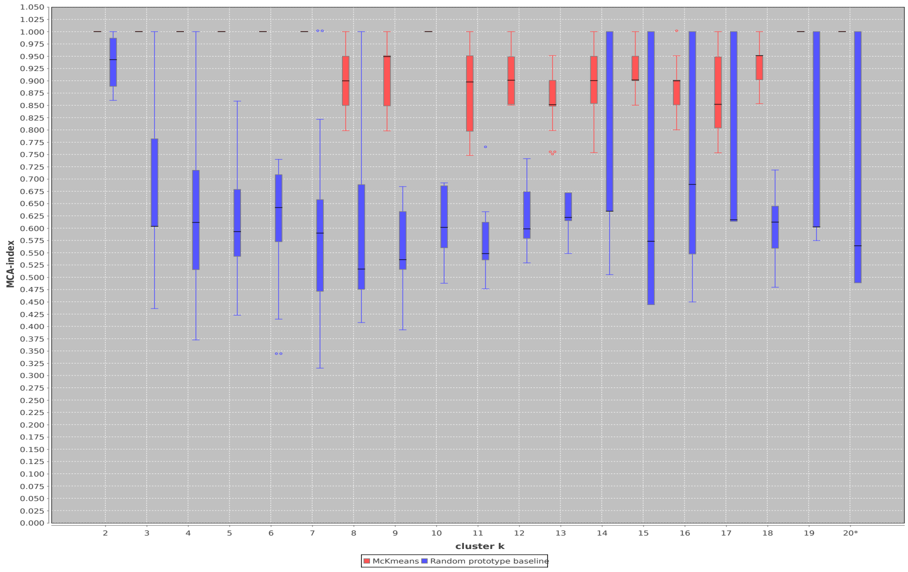

Figure 5: A randomly generated data set (n=50000, d=200, k=20) is resampled 10 times and clustered assuming different number of clusters k. Stability is measured over different numbers of clusters with the MCA index and the random prototype hypothesis. Cluster results are shown as red boxes, baseline results as blue boxes. The best stability (greatest difference between median of cluster results and median of random baseline) is reported for $k = 20$, which is the number of clusters the data set was generated with.

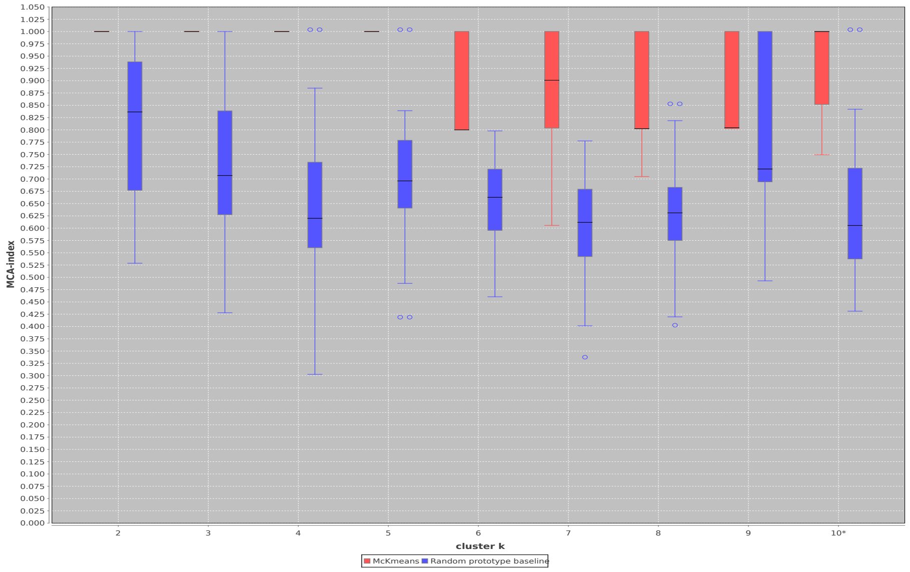

Figure 6: A randomly generated data set (n=50000, d=500, k=10) is resampled 10 times and clustered assuming different number of clusters k. Stability is measured over different numbers of clusters with the MCA index and the random prototype hypothesis. Cluster results are shown as red boxes, baseline results as blue boxes. The best stability (greatest difference between median of cluster results and median of random baseline) is reported for $k = 10$, which is the number of clusters the data set was generated with.

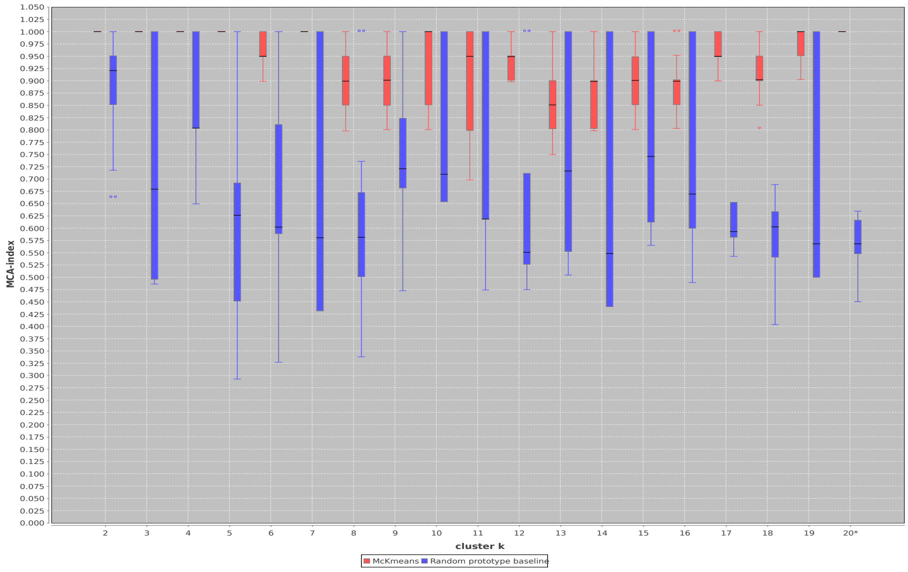

Figure 7: A randomly generated data set (n=50000, d=500, k=20) is resampled 10 times and clustered assuming different number of clusters k. Stability is measured over different numbers of clusters with the MCA index and the random prototype hypothesis. Cluster results are shown as red boxes, baseline results as blue boxes. The best stability (greatest difference between median of cluster results and median of random baseline) is reported for $k = 20$, which is the number of clusters the data set was generated with.

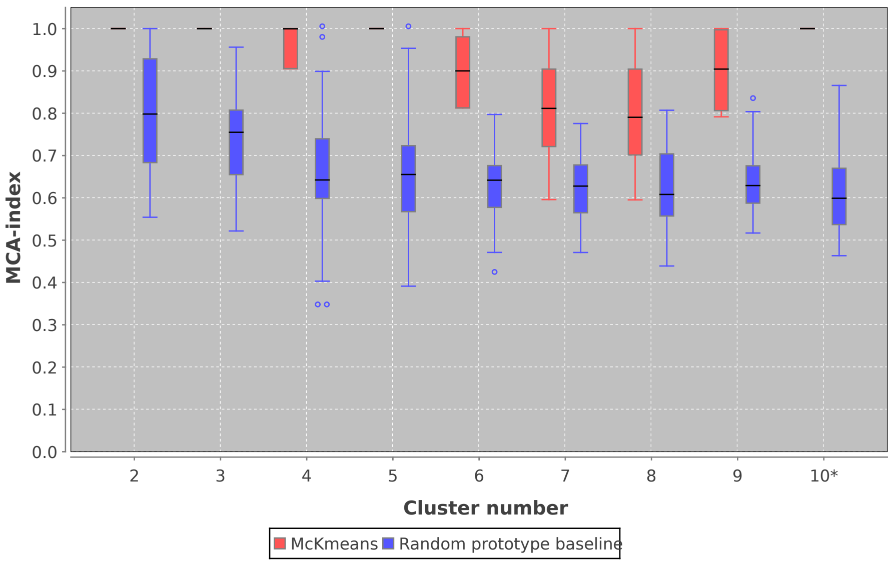

Figure 8: A randomly generated data set (n=100000, d=200, k=10) is resampled 10 times and clustered assuming different number of clusters k. Stability is measured over different numbers of clusters with the MCA index and the random prototype hypothesis. Cluster results are shown as red boxes, baseline results as blue boxes. The best stability (greatest difference between median of cluster results and median of random baseline) is reported for $k = 10$, which is the number of clusters the data set was generated with.

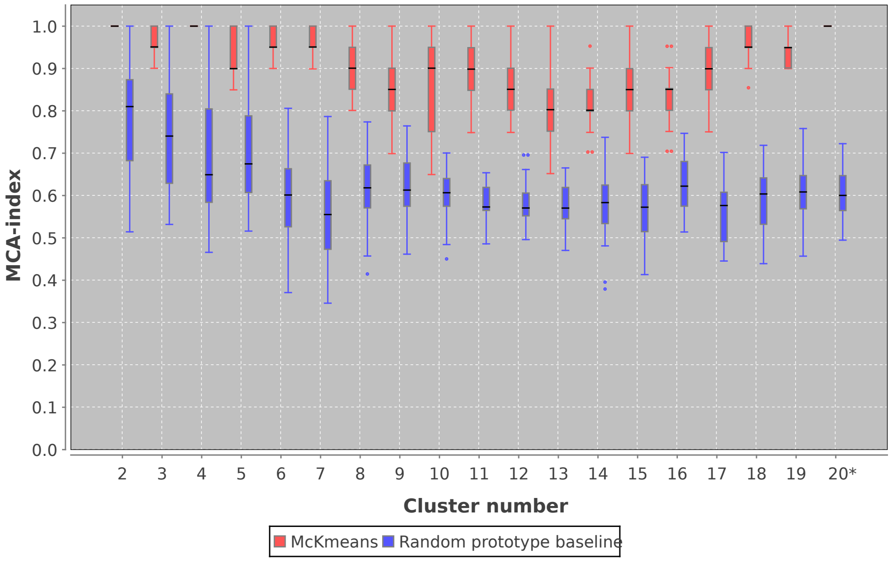

Figure 9: A randomly generated data set (n=100000, d=200, k=20) is resampled 10 times and clustered assuming different number of clusters k. Stability is measured over different numbers of clusters with the MCA index and the random prototype hypothesis. Cluster results are shown as red boxes, baseline results as blue boxes. The best stability (greatest difference between median of cluster results and median of random baseline) is reported for $k = 20$, which is the number of clusters the data set was generated with.

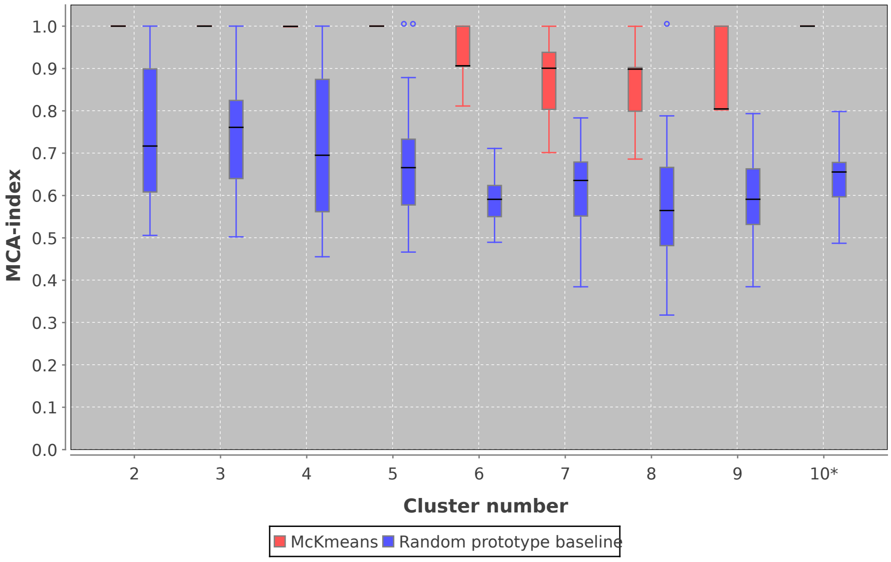

Figure 10: A randomly generated data set (n=100000, d=500, k=10) is resampled 10 times and clustered assuming different number of clusters k. Stability is measured over different numbers of clusters with the MCA index and the random prototype hypothesis. Cluster results are shown as red boxes, baseline results as blue boxes. The best stability (greatest difference between median of cluster results and median of random baseline) is reported for $k = 10$, which is the number of clusters the data set was generated with.

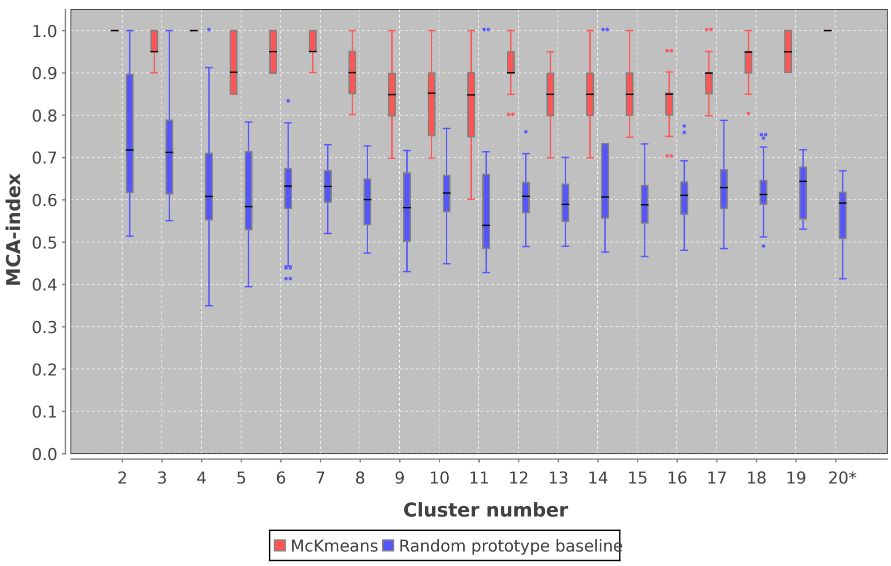

Figure 11: A randomly generated data set (n=100000, d=500, k=20) is resampled 10 times and clustered assuming different number of clusters k. Stability is measured over different numbers of clusters with the MCA index and the random prototype hypothesis. Cluster results are shown as red boxes, baseline results as blue boxes. The best stability (greatest difference between median of cluster results and median of random baseline) is reported for $k = 20$, which is the number of clusters the data set was generated with.
